# Supplementary material for: Transcriptome Analysis of Air Space-Type Variegation Formation in Trifolium pratense
Source: Int J Mol Sci. 2022 Jul 14;23(14):7794. doi: 10.3390/ijms23147794 (PMC9322087; doi:10.3390/ijms23147794)
Supplement: Supplementary file 1 [file ijms-23-07794-s001.zip › ijms-1751573-supplementary.pdf]

International Journal of *Molecular Sciences*

MDPI

Article

Transcriptome Analysis of Air Space Type Variegation Formation in *Trifolium pratense*

Jianhang Zhang, Jiecheng Li, Lu Zou, Hongqing Li*

School of Life Sciences, East China Normal University, Shanghai 200241, China; 52181300002@stu.ecnu.edu.cn (J.Z.); 51201300006@stu.ecnu.edu.cn (J.L.); 15201799715@163.com (L.Z.)

* Correspondence: hqli@bio.ecnu.edu.cn

Table S1 List of DEGs related to the four metabolic pathways of *Trifolium pratense*

Gene ID	Arabidopsis gene name	Arabidopsis orthologs locus	Log_2(RVY_vs_RGY)	Log_2(RV_vs_RG)	Log_2(RV_vs_RVG)	Description	Metabolic pathways
**Cell wall**							
~gene21690	AT3G24480	AT3G24480	1.4	NC	NC	leucine-rich repeat (LRR) family protein (LRR)	cell wall proteins
~gene33514	AT4G29240	AT4G29240	1.2	NC	NC	leucine-rich repeat (LRR) family protein (LRR)	
~gene14364*	RGP2	AT5G15650	2.2	2.8	NC	reversibly glycosylated polypeptide 2 (RGP)	
~gene35890	FLA1	AT5G55730	NC	NC	1.6	FASCICLIN-like arabinogalactan 1 (FLA)	
~gene6444	FLA9	AT1G03870	NC	NC	2.2	FASCICLIN-like arabinogalactan 9 (FLA)	
~gene11538*	FLA11	AT5G03170	1.2	1.8	NC	FASCICLIN-like arabinogalactan-protein 11 (FLA)	
~gene15548	FLA11	AT5G03170	NC	1.1	NC	FASCICLIN-like arabinogalactan-protein 11 (FLA)	
~gene15564*	FLA11	AT5G03170	NC	2.5	1.7	FASCICLIN-like arabinogalactan-protein 11 (FLA)	
~gene4553*	FLA11	AT5G03170	NC	1.3	1.1	FASCICLIN-like arabinogalactan-protein 11 (FLA)	
~gene4564*	FLA11	AT5G03170	NC	1.0	1.4	FASCICLIN-like arabinogalactan-protein 11 (FLA)	
~gene6727	FLA11	AT5G03170	NC	1.5	NC	FASCICLIN-like arabinogalactan-protein 11 (FLA)	
~gene6802*	FLA11	AT5G03170	NC	1.2	1.1	FASCICLIN-like arabinogalactan-protein 11 (FLA)	
~gene11523	FLA12	AT5G60490	NC	1.7	NC	FASCICLIN-like arabinogalactan-protein 12 (FLA)	
~gene15546	FLA12	AT5G60490	NC	1.2	NC	FASCICLIN-like arabinogalactan-protein 12 (FLA)	
~gene39714*	FLA8	AT2G45470	1.1	1.6	NC	FASCICLIN-like arabinogalactan protein 8 (FLA)	

Gene ID	Arabidopsis gene name	Arabidopsis orthologs locus	Log_2(RVY_vs_RGY)	Log_2(RV_vs_RG)	Log_2(RV_vs_RVG)	Description	Metabolic pathways
BGI_novel_G001184*	FLA16	AT2G35860	-5.9	-3.0	NC	FASCICLIN-like arabinogalactan protein 16 precursor (FLA)	
~gene2602	CSLA09	AT5G03760	NC	NC	1.3	nucleotide-diphospho-sugar transferases superfamily protein (CSL)	
~gene28357	CSLA02	AT5G22740	NC	NC	1.4	cellulose synthase-like A02 (CSL)	
~gene5709	CSLA02	AT5G22740	NC	1.4	NC	cellulose synthase-like A02 (CSL)	
~gene5673*	CSLA10	AT1G24070	NC	3.4	1.4	cellulose synthase-like A10 (CSL)	
~gene20922	CSLB03	AT2G32530	-1.9	NC	NC	cellulose synthase-like B3 (CSL)	
~gene29342	CSLB03	AT2G32530	1.4	NC	NC	cellulose synthase-like B3 (CSL)	
~gene4204	CSLB03	AT2G32530	3.7	NC	NC	cellulose synthase-like B3 (CSL)	
~gene33061	CSLC5	AT4G31590	1.3	NC	NC	cellulose-synthase-like C5 (CSL)	cellulose synthesis
~gene19436	CSLD2	AT5G16910	-1.0	NC	NC	cellulose-synthase like D2 (CSL)	
~gene26436	CSLG1	AT4G24010	NC	NC	-1.2	cellulose synthase like G1 (CSL)	
~gene26466	CSLG1	AT4G24010	NC	NC	-1.1	cellulose synthase like G1 (CSL)	
~gene36378	CSLG1	AT4G24010	NC	NC	-1.3	cellulose synthase like G1 (CSL)	
~gene24680	CSLG2	AT4G24000	NC	-1.4	NC	cellulose synthase like G2 (CSL)	
~gene25499	CSLG3	AT4G23990	NC	-2.3	NC	cellulose synthase like G3 (CSL)	
~gene25624	CSLC08	AT2G24630	NC	NC	1.3	glycosyl transferase family 2 protein (CSL)	
~gene24767	CESA10	AT2G25540	NC	1.3	NC	cellulose synthase 10 (CESA)	
~gene3569*	CEV1	AT5G05170	9.0	2.4	NC	cellulose synthase family protein (CESA)	
~gene27971	ASD2	AT5G26120	-1.6	NC	NC	alpha-L-arabinofuranosidase 2 (ASD)	
~gene27539	BGAL2	AT3G52840	-1.1	NC	NC	beta-galactosidase 2 (BGA)	
~gene3757	BGAL2	AT3G52840	NC	1.6	NC	beta-galactosidase 2 (BGA)	
~gene27231	BXL2	AT1G02640	-2.7	NC	NC	beta-xylosidase 2 (BXL)	
~gene27232	BXL2	AT1G02640	-2.9	NC	NC	beta-xylosidase 2 (BXL)	cell wall degradation
~gene23788	PGIP1	AT5G06860	3.5	NC	NC	polygalacturonase inhibiting protein 1 (PGIP)	
~gene23837*	PGIP1	AT5G06860	1.5	2.1	NC	polygalacturonase inhibiting protein 1 (PGIP)	
~gene23143	PGIP2	AT5G06870	7.0	NC	-3.4	polygalacturonase inhibiting protein 2 (PGIP)	
~gene23146	PGIP2	AT5G06870	NC	NC	1.4	polygalacturonase inhibiting protein 2 (PGIP)	
~gene23150	PGIP2	AT5G06870	NC	NC	1.2	polygalacturonase inhibiting protein 2 (PGIP)	

**Gene ID**	**Arabidopsis gene name**	**Arabidopsis orthologs locus**	**Log2(RVY_vs_RGY)**	**Log2(RV_vs_RG)**	**Log2(RV_vs_RVG)**	**Description**	**Metabolic pathways**
~gene23816	PGIP2	AT5G06870	NC	-1.4	NC	polygalacturonase inhibiting protein 2 (PGIP)	
~gene39861	PGIP2	AT5G06870	7.9	NC	-3.7	polygalacturonase inhibiting protein 2 (PGIP)	
~gene11671*	PG3	AT1G23760	1.0	1.3	2.2	polygalacturonase family (PG)	
~gene30808	AT1G02460	AT1G02460	1.1	NC	NC	pectin lyase-like superfamily protein (PLL)	
~gene9533*	AT1G10640	AT1G10640	NC	1.1	1.4	pectin lyase-like superfamily protein (PLL)	
~gene100	AT3G06770	AT3G06770	-1.9	NC	NC	pectin lyase-like superfamily protein (PLL)	
~gene26302	PMR6	AT3G54920	NC	NC	1.2	pectin lyase-like superfamily protein (PLL)	
~gene12808	AT3G61490	AT3G61490	NC	NC	2.1	pectin lyase-like superfamily protein (PLL)	
~gene142	AT3G61490	AT3G61490	4.3	NC	NC	pectin lyase-like superfamily protein (PLL)	
~gene31748	AT2G36710	AT2G36710	NC	NC	5.6	pectin lyase-like superfamily protein (PLL)	
~gene25565*	AT5G19730	AT5G19730	1.9	1.5	NC	pectin lyase-like superfamily protein (PLL)	
~gene4140	AT4G01890	AT4G01890	1.2	NC	NC	pectin lyase-like superfamily protein (PLL)	
~gene19423*	AT4G23500	AT4G23500	NC	2.2	1.6	pectin lyase-like superfamily protein (PLL)	
~gene40029	AT4G23500	AT4G23500	NC	1.5	NC	pectin lyase-like superfamily protein (PLL)	
~gene21012	AT5G04310	AT5G04310	1.4	NC	NC	pectin lyase-like superfamily protein (PLL)	
~gene23278	AT5G48900	AT5G48900	NC	NC	1.5	pectin lyase-like superfamily protein (PLL)	
~gene20617	AT5G63180	AT5G63180	1.2	NC	NC	pectin lyase-like superfamily protein (PLL)	
~gene21640	AT5G63180	AT5G63180	1.1	NC	NC	pectin lyase-like superfamily protein (PLL)	
~gene38600	AT5G63180	AT5G63180	1.2	NC	NC	pectin lyase-like superfamily protein (PLL)	
~gene187	AT1G09890	AT1G09890	NC	NC	1.1	rhamnogalacturonate lyase family protein (RG)	
~gene30292	GH9B5	AT1G19940	NC	NC	1.1	glycosyl hydrolase 9B5 (GH)	
~gene27081	AT4G33830	AT4G33830	-1.5	NC	NC	glycosyl hydrolase family 10 protein (GH)	
~gene35768	CEL3	AT1G71380	1.4	NC	NC	cellulase 3 (CEL)	
~gene27531	AT5G03795	AT5G03795	NC	5.0	NC	exostosin family protein (EXT)	hemicellulose synthesis
~gene21038	F8H	AT5G22940	NC	-1.2	1.0	FRA8 homolog (GT)	
~gene40180	AT1G74800	AT1G74800	1.7	NC	NC	galactosyltransferase family protein (GAL)	
~gene11949*	EXPA1	AT1G69530	NC	2.0	1.8	expansin A1 (EXP)	cell wall modification
~gene33611*	EXPA1	AT1G69530	NC	1.4	2.6	expansin A1 (EXP)	
~gene26690	EXPA16	AT3G55500	NC	-1.5	NC	expansin A16 (EXP)	
~gene5808	EXPA2	AT5G05290	NC	NC	1.0	expansin A2 (EXP)	

Gene ID	Arabidopsis gene name	Arabidopsis orthologs locus	Log_2(RVY_vs_RGY)	Log_2(RV_vs_RG)	Log_2(RV_vs_RVG)	Description	Metabolic pathways
~gene5820	EXPA2	AT5G05290	NC	1.1	NC	expansin A2 (EXP)	
~gene34310	EXPA4	AT2G39700	1.1	NC	NC	expansin A4 (EXP)	
~gene12731	EXPA9	AT5G02260	2.9	NC	NC	expansin A9 (EXP)	
~gene21756	EXPA9	AT5G02260	1.5	NC	NC	expansin A9 (EXP)	
~gene6476	EXPB1	AT2G20750	NC	-2.5	NC	expansin B1 (EXP)	
~gene21258	EXLA1	AT3G45970	-1.2	NC	NC	expansin-like A1 (EXLA)	
~gene31424	EXLA2	AT4G38400	1.5	NC	2.4	expansin-like A2 (EXLA)	
~gene31190	XTH31	AT3G44990	2.1	NC	NC	xyloglucan endo-transglycosylase-related 8 (XTH)	
~gene32828	XTH31	AT3G44990	2.1	NC	NC	xyloglucan endo-transglycosylase-related 8 (XTH)	
~gene7390*	XTH31	AT3G44990	NC	2.4	2.3	xyloglucan endo-transglycosylase-related 8 (XTH)	
~gene14522	XTH1	AT4G13080	NC	1.5	NC	xyloglucan endotransglucosylase/hydrolase 1 (XTH)	
~gene9322	XTH15	AT4G14130	1.2	NC	NC	xyloglucan endotransglucosylase/hydrolase 15 (XTH)	
~gene23492	XTH16	AT3G23730	2.0	NC	NC	xyloglucan endotransglucosylase/hydrolase 16 (XTH)	
~gene27952	XTH16	AT3G23730	1.0	NC	NC	xyloglucan endotransglucosylase/hydrolase 16 (XTH)	
~gene19435	XTH29	AT4G18990	1.2	NC	NC	xyloglucan endotransglucosylase/hydrolase 29 (XTH)	
~gene31657	XTH4	AT2G06850	NC	NC	2.3	xyloglucan endotransglucosylase/hydrolase 4 (XTH)	
~gene6084	XTH4	AT2G06850	NC	1.0	NC	xyloglucan endotransglucosylase/hydrolase 4 (XTH)	
~gene5465	XTH7	AT4G37800	NC	NC	1.8	xyloglucan endotransglucosylase/hydrolase 7 (XTH)	
~gene11467	XTH22	AT5G57560	NC	-1.5	1.2	xyloglucan endotransglucosylase/hydrolase family protein (XTH)	
~gene14039	XTH22	AT5G57560	NC	NC	2.8	xyloglucan endotransglucosylase/hydrolase family protein (XTH)	

Gene ID	Arabidopsis gene name	Arabidopsis orthologs locus	Log_2(RVY_vs_RGY)	Log_2(RV_vs_RG)	Log_2(RV_vs_RVG)	Description	Metabolic pathways
~gene35463	XTH22	AT5G57560	NC	NC	2.5	xyloglucan endotransglucosylase/hydrolase family protein (XTH)	
~gene5284	XTH22	AT5G57560	NC	NC	2.0	xyloglucan endotransglucosylase/hydrolase family protein (XTH)	
~gene5325	XTH22	AT5G57560	NC	-1.7	NC	xyloglucan endotransglucosylase/hydrolase family protein (XTH)	
~gene5356	XTH22	AT5G57560	NC	-1.3	NC	xyloglucan endotransglucosylase/hydrolase family protein (XTH)	
~gene5432	XTH22	AT5G57560	1.8	NC	NC	xyloglucan endotransglucosylase/hydrolase family protein (XTH)	
~gene34569	EXGT-A3	AT2G01850	1.3	NC	1.1	endoxyloglucan transferase A3 (EXGT)	
BGI_novel_G000197	GAUT3	AT4G38270	-1.8	NC	NC	galacturonosyltransferase 3 (GAUT)	pectin synthesis
~gene26837	SKU5	AT4G12420	NC	-1.1	NC	cupredoxin superfamily protein (SKU)	
~gene16820	AT4G19410	AT4G19410	2.0	NC	3.7	pectinacetylesterase family protein (PAE)	
~gene30314	AT4G19410	AT4G19410	NC	1.0	NC	pectinacetylesterase family protein (PAE)	
~gene28957	SKS2	AT5G51480	NC	1.0	NC	SKU5 similar 2 (SKS)	
~gene31499*	SKS2	AT5G51480	2.9	3.9	NC	SKU5 similar 2 (SKS)	
~gene31503*	SKS2	AT5G51480	1.1	1.3	NC	SKU5 similar 2 (SKS)	
~gene26393	SKS6	AT1G41830	NC	NC	1.5	SKU5-similar 6 (SKS)	
~gene38201*	SKS6	AT1G41830	NC	2.2	2.7	SKU5-similar 6 (SKS)	
~gene19383*	PMEPCRF	AT5G53370	NC	1.1	3.4	pectin methylesterase PCR fragment F (PME)	pectin * esterases
~gene9600	AT1G02810	AT1G02810	1.0	NC	NC	plant invertase/pectin methylesterase inhibitor superfamily (PME)	
~gene10172*	PME19	AT1G11590	1.1	1.4	NC	plant invertase/pectin methylesterase inhibitor superfamily (PME)	
~gene10180*	PME19	AT1G11590	NC	1.8	1.9	plant invertase/pectin methylesterase inhibitor superfamily (PME)	
~gene11123*	PME19	AT1G11590	1.7	1.5	1.7	plant invertase/pectin methylesterase inhibitor superfamily (PME)	

Gene ID	Arabidopsis gene name	Arabidopsis orthologs locus	Log_2(RVY_vs_RGY)	Log_2(RV_vs_RG)	Log_2(RV_vs_RVG)	Description	Metabolic pathways
~gene22105*	PME19	AT1G11590	1.2	1.8	NC	plant invertase/pectin methylesterase inhibitor superfamily (PME)	
~gene26807	PME19	AT1G11590	2.0	NC	NC	plant invertase/pectin methylesterase inhibitor superfamily (PME)	
~gene26845*	PME19	AT1G11590	1.8	1.9	1.0	plant invertase/pectin methylesterase inhibitor superfamily (PME)	
~gene32680	AT2G45220	AT2G45220	NC	1.9	NC	plant invertase/pectin methylesterase inhibitor superfamily (PME)	
~gene21781	AT3G10720	AT3G10720	-1.2	NC	1.1	plant invertase/pectin methylesterase inhibitor superfamily (PME)	
~gene5092	AT3G10720	AT3G10720	-1.7	NC	1.0	plant invertase/pectin methylesterase inhibitor superfamily (PME)	
~gene1603	AT3G43270	AT3G43270	NC	NC	2.4	plant invertase/pectin methylesterase inhibitor superfamily (PME)	
~gene3031*	AT5G27870	AT5G27870	1.4	1.6	NC	plant invertase/pectin methylesterase inhibitor superfamily (PME)	
~gene7588	AT5G51490	AT5G51490	7.9	NC	NC	plant invertase/pectin methylesterase inhibitor superfamily (PME)	
~gene7596*	AT5G51490	AT5G51490	NC	1.3	1.6	plant invertase/pectin methylesterase inhibitor superfamily (PME)	
~gene25461	GALAK	AT3G10700	NC	NC	1.3	encodes a GHMP kinase family protein (GALK)	
~gene2647	GALAK	AT3G10700	NC	NC	1.4	encodes a GHMP kinase family protein (GALK)	
~gene31950	GALK2	AT5G14470	-1.0	NC	NC	GHMP kinase family protein (GALK)	
~gene8010	AT4G20460	AT4G20460	1.1	NC	NC	NAD(P)-binding Rossmann-fold superfamily protein (NADP)	precursor synthesis
~gene5954	UGE3	AT1G63180	NC	1.2	NC	UDP-D-glucose/UDP-D-galactose 4-epimerase 3 (UGE)	
~gene10970	RHM3	AT3G14790	-1.0	NC	NC	rhamnose biosynthesis 3 (RHM)	
**Photosystem**							
~gene2535	LHCB4.2	AT3G08940	-1.1	NC	NC	light harvesting complex photosystem II (LHCB)	lightreaction

Gene ID	Arabidopsis gene name	Arabidopsis orthologs locus	Log_2(RVY_vs_RGY)	Log_2(RV_vs_RG)	Log_2(RV_vs_RVG)	Description	Metabolic pathways
~gene32852	LHCB2.1	AT2G05100	-1.3	NC	NC	photosystem II light harvesting complex gene 2.1 (LHCB)	
~gene1283	LHCB2.2	AT2G05070	-1.0	NC	NC	photosystem II light harvesting complex gene 2.2 (LHCB)	
~gene27011*	LHCB1.3	AT1G29930	-2.6	-1.8	NC	chlorophyll A/B binding protein 1 (LHCB)	
~gene27016*	LHCB1.3	AT1G29930	-1.9	-1.8	NC	chlorophyll A/B binding protein 1 (LHCB)	
~gene4884	LHCB1.3	AT1G29930	-1.2	NC	NC	chlorophyll A/B binding protein 1 (LHCB)	
~gene18594	LHCB1.2	AT1G29910	-1.2	NC	NC	chlorophyll A/B binding protein 3 (LHCB)	
~gene21404*	LHCB1.2	AT1G29910	-1.7	-1.4	NC	chlorophyll A/B binding protein 3 (LHCB)	
~gene26972*	LHCB1.2	AT1G29910	-2.1	-1.2	NC	chlorophyll A/B binding protein 3 (LHCB)	
~gene26997*	LHCB1.2	AT1G29910	-1.3	-1.9	1.0	chlorophyll A/B binding protein 3 (LHCB)	
~gene38654	CP12-1	AT2G47400	-1.2	NC	NC	CP12 domain-containing protein 1 (CP12)	
~gene15829	AT1G73110	AT1G73110	-1.4	NC	NC	P-loop containing nucleoside triphosphate hydrolases superfamily protein (P-loop)	
BGI_novel_G000242	RPE	AT5G61410	-1.1	NC	NC	aldolase-type TIM barrel family protein (RPE)	
~gene9091	UKL5	AT3G27440	-1.1	NC	NC	uridine kinase-like 5 (UKL)	
BGI_novel_G001418*	RBCS1B	AT5G38430	-3.1	-1.5	NC	ribulose bisphosphate carboxylase (small chain) family protein (RBCS)	
BGI_novel_G001419	RBCS1B	AT5G38430	NC	1.7	NC	ribulose bisphosphate carboxylase (small chain) family protein (RBCS)	calvin cycle
~gene40847	RBCS2B	AT5G38420	-1.1	NC	NC	ribulose bisphosphate carboxylase (small chain) family protein (RBCS)	
~gene20656	GAPA	AT3G26650	-1.0	NC	NC	glyceraldehyde 3-phosphate dehydrogenase A subunit (GAP)	
~gene34873	FBP	AT1G43670	-1.2	NC	NC	Inositol monophosphatase family protein (FBP)	
~gene29802	FBP	AT1G43670	-1.1	NC	NC	Inositol monophosphatase family protein (FBP)	
BGI_novel_G001124	FBA7	AT4G26520	-1.4	NC	NC	aldolase superfamily protein (FBA)	
~gene31327	AT3G60750	AT3G60750	1.3	NC	NC	transketolase (TKL)	
~gene11070	AT2G45630	AT2G45630	2.4	NC	NC	D-isomer specific 2-hydroxyacid dehydrogenase family protein (HPPR)	

Gene ID	Arabidopsis gene name	Arabidopsis orthologs locus	Log_2(RVY_vs_RGY)	Log_2(RV_vs_RG)	Log_2(RV_vs_RVG)	Description	Metabolic pathways
~gene32959	SHM6	AT1G22020	NC	NC	-1.0	serine hydroxymethyltransferase 6 (SHM)	photorespiration
~gene29765	GOX2	AT3G14415	-1.1	NC	NC	aldolase-type TIM barrel family protein (GOX)	
~gene9583*	GOX2	AT3G14415	3.8	2.2	NC	aldolase-type TIM barrel family protein (GOX)	
~gene9584*	GOX2	AT3G14415	1.6	2.3	NC	aldolase-type TIM barrel family protein (GOX)	
~gene36085	GLDP1	AT4G33010	-1.3	NC	NC	glycine decarboxylase P-protein 1 (GLDP)	
~gene38783	AOAT1	AT1G23310	-1.0	NC	NC	glutamate:glyoxylate aminotransferase (AOAT)	
~gene19354*	AGT	AT2G13360	1.8	2.4	6.0	encodes a peroxisomal photorespiratory enzyme (AGT)	
~gene8487	AGT	AT2G13360	-1.1	NC	NC	encodes a peroxisomal photorespiratory enzyme (AGT)	
~gene18144	AT4G17360	AT4G17360	-3.2	NC	NC	formyl transferase (AT4G17360)	
**Redox**							
~gene32128	AT1G06640	AT1G06640	1.1	NC	NC	2-oxoglutarate (2OG) and Fe(II)-dependent oxygenase superfamily protein (2OG)	
~gene11912	AT3G19010	AT3G19010	NC	2.0	NC	2-oxoglutarate (2OG) and Fe(II)-dependent oxygenase superfamily protein (2OG)	
~gene23506	AT3G19010	AT3G19010	NC	NC	-1.1	2-oxoglutarate (2OG) and Fe(II)-dependent oxygenase superfamily protein (2OG)	
~gene23520	AT3G19010	AT3G19010	NC	-1.6	NC	2-oxoglutarate (2OG) and Fe(II)-dependent oxygenase superfamily protein (2OG)	
BGI_novel_G000042*	AT3G61400	AT3G61400	1.8	1.6	NC	2-oxoglutarate (2OG) and Fe(II)-dependent oxygenase superfamily protein (2OG)	ascorbate and glutathione
BGI_novel_G000043*	AT3G61400	AT3G61400	1.4	1.4	NC	2-oxoglutarate (2OG) and Fe(II)-dependent oxygenase superfamily protein (2OG)	
BGI_novel_G000044*	AT3G61400	AT3G61400	4.0	3.2	NC	2-oxoglutarate (2OG) and Fe(II)-dependent oxygenase superfamily protein (2OG)	
BGI_novel_G000046	AT3G61400	AT3G61400	NC	1.0	NC	2-oxoglutarate (2OG) and Fe(II)-dependent oxygenase superfamily protein (2OG)	
BGI_novel_G000048	AT3G61400	AT3G61400	NC	1.2	NC	2-oxoglutarate (2OG) and Fe(II)-dependent oxygenase superfamily protein (2OG)	

Gene ID	Arabidopsis gene name	Arabidopsis orthologs locus	Log_2(RVY_vs_RGY)	Log_2(RV_vs_RG)	Log_2(RV_vs_RVG)	Description	Metabolic pathways
BGI_novel_G000357	AT3G61400	AT3G61400	NC	1.0	NC	2-oxoglutarate (2OG) and Fe(II)-dependent oxygenase superfamily protein (2OG)	
BGI_novel_G001050	AT3G61400	AT3G61400	NC	1.1	NC	2-oxoglutarate (2OG) and Fe(II)-dependent oxygenase superfamily protein (2OG)	
~gene16545	AT3G61400	AT3G61400	1.2	NC	NC	2-oxoglutarate (2OG) and Fe(II)-dependent oxygenase superfamily protein (2OG)	
~gene31276	AT3G61400	AT3G61400	-2.5	NC	NC	2-oxoglutarate (2OG) and Fe(II)-dependent oxygenase superfamily protein (2OG)	
~gene36936	AT5G59540	AT5G59540	-2.7	NC	NC	2-oxoglutarate (2OG) and Fe(II)-dependent oxygenase superfamily protein (2OG)	
BGI_novel_G001279	GSH1	AT4G23100	NC	-1.7	NC	encodes the enzyme glutamate-cysteine ligase catalyzing the first (GSH)	
BGI_novel_G001280	GSH1	AT4G23100	NC	-1.3	NC	encodes the enzyme glutamate-cysteine ligase catalyzing the first (GSH)	
~gene12862	GSH1	AT4G23100	NC	NC	-1.3	encodes the enzyme glutamate-cysteine ligase catalyzing the first (GSH)	
~gene30225	GSH1	AT4G23100	1.4	NC	NC	encodes the enzyme glutamate-cysteine ligase catalyzing the first (GSH)	
~gene421	GSH1	AT4G23100	1.1	NC	NC	encodes the enzyme glutamate-cysteine ligase catalyzing the first (GSH)	
~gene6641*	GSH1	AT4G23100	6.4	9.4	NC	encodes the enzyme glutamate-cysteine ligase catalyzing the first (GSH)	
~gene33164	GSH2	AT5G27380	NC	NC	-1.7	encodes a protein with similarity to glutathione synthetases (GSH)	
~gene21072	GGT1	AT4G39640	1.8	NC	NC	gamma-glutamyl transpeptidase 1 (GGT)	
~gene345	APX5	AT4G35970	NC	NC	4.2	ascorbate peroxidase 5 (APX)	
~gene4834	AT2G30890	AT2G30890	1.4	NC	NC	cytochrome b561/ferric reductase transmembrane protein family (Cyt_b561)	
~gene5096	ACYB-2	AT4G25570	NC	NC	2.9	cytochrome b561/ferric reductase transmembrane protein family (Cyt_b561)	
**N-metabolism**							

Gene ID	Arabidopsis gene name	Arabidopsis orthologs locus	Log_2(RVY_vs_RGY)	Log_2(RV_vs_RG)	Log_2(RV_vs_RVG)	Description	Metabolic pathways
~gene34622	GDH1	AT5G18170	1.0	NC	-2.2	glutamate dehydrogenase 1 (GDH)	N-degradation
~gene34624	GDH3	AT3G03910	1.3	NC	-2.0	glutamate dehydrogenase 3 (GDH)	
~gene30627	GLU2	AT2G41220	-1.2	NC	NC	glutamate synthase 2 (GLU)	
~gene18383	GSR2	AT1G66200	NC	1.1	NC	glutamine synthase clone F11 (GSR)	
~gene24906	GSR1	AT5G37600	NC	1.0	NC	glutamine synthase clone R1 (GSR)	ammonia metabolism
~gene21005	NodGS	AT3G53180	1.2	NC	NC	glutamate-ammonia ligases (NodGS)	
~gene21013*	NodGS	AT3G53180	3.8	1.2	NC	glutamate-ammonia ligases (NodGS)	
~gene30717	NodGS	AT3G53180	1.9	NC	NC	glutamate-ammonia ligases (NodGS)	
~gene8508	NIA1	AT1G77760	1.3	NC	NC	nitrate reductase 1 (NIA)	nitrate metabolism

Note: NC, Log_2(Fold change) <|1| deemed no significant difference. *, candidate genes associated with leaf variegation of Trifolium pratense.

Table S2 List of DEGs related to ubiquitin and autophagy dependent degradation

Gene ID	Arabidopsis orthologs locus	Log_2(RVY_vs_RGY)	Log_2(RV_vs_RG)	Log_2(RV_vs_RVG)	BinName
Tp57577_TGAC_v2_gene14469	AT3G46460	1.0	NC	NC	protein.degradation.ubiquitin.E2
Tp57577_TGAC_v2_gene9731	AT1G53025	6.2	NC	NC	protein.degradation.ubiquitin.E2
Tp57577_TGAC_v2_gene26175	AT5G46210	NC	-1.6	NC	protein.degradation.ubiquitin.E3.APC
Tp57577_TGAC_v2_gene4955	AT4G21100	1.3	1.8	NC	protein.degradation.ubiquitin.E3.DCX
Tp57577_TGAC_v2_gene31110	AT4G38600	1.2	NC	NC	protein.degradation.ubiquitin.E3.HECT
Tp57577_TGAC_v2_gene26230	AT1G04020	1.4	NC	NC	protein.degradation.ubiquitin.E3.RING
Tp57577_TGAC_v2_gene155	AT5G27420	1.5	NC	NC	protein.degradation.ubiquitin.E3.RING
Tp57577_TGAC_v2_gene37898	AT4G34850	1.6	1.1	NC	protein.degradation.ubiquitin.E3.RING
Tp57577_TGAC_v2_gene36787	AT5G25560	-2.1	NC	NC	protein.degradation.ubiquitin.E3.RING
Tp57577_TGAC_v2_gene10302	AT3G11130	1.3	1.4	NC	protein.degradation.ubiquitin.E3.RING
Tp57577_TGAC_v2_gene9071	AT5G40720	1.4	NC	NC	protein.degradation.ubiquitin.E3.RING

Gene ID	Arabidopsis orthologs locus	Log_2(RVY_ vs_RGY)	Log_2(RV_ vs_RG)	Log_2(RV_ vs_RVG)	BinName
Tp57577_TGAC_v2_gene652	AT3G46510	NC	-1.3	NC	protein.degradation.ubiquitin.E3.RING
Tp57577_TGAC_v2_gene5018	AT1G29340	NC	NC	-1.3	protein.degradation.ubiquitin.E3.RING
Tp57577_TGAC_v2_gene13587	AT1G10560	1.0	NC	NC	protein.degradation.ubiquitin.E3.RING
Tp57577_TGAC_v2_gene35031	AT1G22670	1.1	1.1	NC	protein.degradation.ubiquitin.E3.RING
Tp57577_TGAC_v2_gene1104	AT3G01650	-1.2	NC	NC	protein.degradation.ubiquitin.E3.RING
BGI_novel_G000163	AT3G01650	NC	NC	-2.1	protein.degradation.ubiquitin.E3.RING
Tp57577_TGAC_v2_gene40742	AT5G14420	NC	NC	-1.0	protein.degradation.ubiquitin.E3.RING
Tp57577_TGAC_v2_gene19774	AT4G02075	2.3	NC	NC	protein.degradation.ubiquitin.E3.RING
Tp57577_TGAC_v2_gene4028	AT1G02610	1.4	NC	NC	protein.degradation.ubiquitin.E3.RING
BGI_novel_G001025	AT4G26580	-1.4	NC	NC	protein.degradation.ubiquitin.E3.RING
Tp57577_TGAC_v2_gene10181	AT3G19910	1.2	1.6	NC	protein.degradation.ubiquitin.E3.RING
Tp57577_TGAC_v2_gene14688	AT3G53690	2.1	NC	NC	protein.degradation.ubiquitin.E3.RING
Tp57577_TGAC_v2_gene24399	AT2G25410	1.2	NC	NC	protein.degradation.ubiquitin.E3.RING
Tp57577_TGAC_v2_gene26090	AT2G15580	-1.2	NC	NC	protein.degradation.ubiquitin.E3.RING
Tp57577_TGAC_v2_gene2876	AT1G72220	1.1	NC	NC	protein.degradation.ubiquitin.E3.RING
Tp57577_TGAC_v2_gene28868	AT1G53820	3.0	NC	-1.4	protein.degradation.ubiquitin.E3.RING
Tp57577_TGAC_v2_gene32136	AT1G72200	1.9	NC	NC	protein.degradation.ubiquitin.E3.RING
Tp57577_TGAC_v2_gene35394	AT5G03200	1.5	NC	NC	protein.degradation.ubiquitin.E3.RING
Tp57577_TGAC_v2_gene37357	AT1G49200	-1.1	-1.0	1.5	protein.degradation.ubiquitin.E3.RING
Tp57577_TGAC_v2_gene400	AT4G26580	-1.5	NC	NC	protein.degradation.ubiquitin.E3.RING
Tp57577_TGAC_v2_gene590	AT2G46495	1.4	NC	NC	protein.degradation.ubiquitin.E3.RING
Tp57577_TGAC_v2_gene6239	AT4G33565	1.0	1.5	NC	protein.degradation.ubiquitin.E3.RING
Tp57577_TGAC_v2_gene6364	AT2G20030	1.3	NC	NC	protein.degradation.ubiquitin.E3.RING
Tp57577_TGAC_v2_gene7433	AT3G10910	1.9	NC	NC	protein.degradation.ubiquitin.E3.RING
Tp57577_TGAC_v2_gene24379	AT2G46495	NC	1.5	NC	protein.degradation.ubiquitin.E3.RING

Gene ID	Arabidopsis orthologs locus	Log_2(RVY_ vs_RGY)	Log_2(RV_ vs_RG)	Log_2(RV_ vs_RVG)	BinName
Tp57577_TGAC_v2_gene5489	AT3G14250	NC	1.0	NC	protein.degradation.ubiquitin.E3.RING
Tp57577_TGAC_v2_gene6413	AT2G42360	NC	3.0	NC	protein.degradation.ubiquitin.E3.RING
Tp57577_TGAC_v2_gene18146	AT3G61550	NC	NC	-1.2	protein.degradation.ubiquitin.E3.RING
Tp57577_TGAC_v2_gene26380	AT1G49220	NC	NC	1.3	protein.degradation.ubiquitin.E3.RING
Tp57577_TGAC_v2_gene8112	AT3G14250	NC	NC	-1.4	protein.degradation.ubiquitin.E3.RING
Tp57577_TGAC_v2_gene19186	AT4G17680	1.4	NC	NC	protein.degradation.ubiquitin.E3.RING
Tp57577_TGAC_v2_gene9391	AT3G12920	4.3	NC	NC	protein.degradation.ubiquitin.E3.RING
Tp57577_TGAC_v2_gene33935	AT4G17680	NC	NC	-1.0	protein.degradation.ubiquitin.E3.RING
Tp57577_TGAC_v2_gene14398	AT1G02860	NC	1.1	NC	protein.degradation.ubiquitin.E3.RING
Tp57577_TGAC_v2_gene33957	AT2G38920	NC	NC	-1.1	protein.degradation.ubiquitin.E3.RING
Tp57577_TGAC_v2_gene9681	AT4G02110	1.4	NC	NC	protein.degradation.ubiquitin.E3.RING
Tp57577_TGAC_v2_gene21299	AT1G11950	1.1	NC	NC	protein.degradation.ubiquitin.E3.RING
Tp57577_TGAC_v2_gene33056	AT1G11950	2.2	NC	NC	protein.degradation.ubiquitin.E3.RING
Tp57577_TGAC_v2_gene32811	AT5G03750	1.0	NC	NC	protein.degradation.ubiquitin.E3.RING
Tp57577_TGAC_v2_gene3475	AT4G27240	7.5	9.1	9.2	protein.degradation.ubiquitin.E3.RING
Tp57577_TGAC_v2_gene23473	AT1G57800	1.1	NC	NC	protein.degradation.ubiquitin.E3.RING
Tp57577_TGAC_v2_gene5619	AT3G46620	1.0	NC	NC	protein.degradation.ubiquitin.E3.RING
Tp57577_TGAC_v2_gene15269	AT2G22680	NC	NC	1.4	protein.degradation.ubiquitin.E3.RING
Tp57577_TGAC_v2_gene8712	AT5G06420	1.1	NC	NC	protein.degradation.ubiquitin.E3.RING
Tp57577_TGAC_v2_gene7056	AT1G79110	2.7	2.0	NC	protein.degradation.ubiquitin.E3.RING
Tp57577_TGAC_v2_gene8360	AT5G22660	NC	-1.8	NC	protein.degradation.ubiquitin.E3.SCF.FBOX
Tp57577_TGAC_v2_gene35731	AT3G54650	1.5	NC	NC	protein.degradation.ubiquitin.E3.SCF.FBOX
Tp57577_TGAC_v2_gene29403	AT4G12560	1.4	NC	NC	protein.degradation.ubiquitin.E3.SCF.FBOX
Tp57577_TGAC_v2_gene3366	AT4G12560	NC	1.2	NC	protein.degradation.ubiquitin.E3.SCF.FBOX
BGI_novel_G000231	AT1G32600	-1.2	2.0	NC	protein.degradation.ubiquitin.E3.SCF.FBOX

Gene ID	Arabidopsis orthologs locus	Log_2(RVY_ vs_RGY)	Log_2(RV_ vs_RG)	Log_2(RV_ vs_RVG)	BinName
Tp57577_TGAC_v2_gene7772	AT5G52880	1.3	NC	NC	protein.degradation.ubiquitin.E3.SCF.FBOX
Tp57577_TGAC_v2_gene10820	AT2G02240	2.1	1.2	NC	protein.degradation.ubiquitin.E3.SCF.FBOX
Tp57577_TGAC_v2_gene1238	AT4G24210	2.0	NC	NC	protein.degradation.ubiquitin.E3.SCF.FBOX
Tp57577_TGAC_v2_gene16157	AT3G56470	1.5	1.8	NC	protein.degradation.ubiquitin.E3.SCF.FBOX
Tp57577_TGAC_v2_gene18046	AT3G06240	1.6	NC	NC	protein.degradation.ubiquitin.E3.SCF.FBOX
Tp57577_TGAC_v2_gene23915	AT3G06240	4.6	3.9	NC	protein.degradation.ubiquitin.E3.SCF.FBOX
Tp57577_TGAC_v2_gene24388	AT2G32560	1.3	NC	NC	protein.degradation.ubiquitin.E3.SCF.FBOX
Tp57577_TGAC_v2_gene33673	AT3G06240	1.1	1.2	NC	protein.degradation.ubiquitin.E3.SCF.FBOX
BGI_novel_G000785	AT3G06240	NC	-1.2	NC	protein.degradation.ubiquitin.E3.SCF.FBOX
BGI_novel_G001057	AT3G06240	NC	-5.9	NC	protein.degradation.ubiquitin.E3.SCF.FBOX
Tp57577_TGAC_v2_gene5266	AT5G27920	NC	1.1	NC	protein.degradation.ubiquitin.E3.SCF.FBOX
Tp57577_TGAC_v2_gene32423	AT4G21510	NC	NC	-1.7	protein.degradation.ubiquitin.E3.SCF.FBOX
BGI_novel_G001090	AT2G17036	-2.0	NC	NC	protein.degradation.ubiquitin.E3.SCF.FBOX
BGI_novel_G001093	AT2G17036	-1.5	-2.0	NC	protein.degradation.ubiquitin.E3.SCF.FBOX
Tp57577_TGAC_v2_gene35299	AT2G17036	NC	-1.2	NC	protein.degradation.ubiquitin.E3.SCF.FBOX
Tp57577_TGAC_v2_gene2126	AT1G67190	1.1	NC	NC	protein.degradation.ubiquitin.E3.SCF.FBOX
Tp57577_TGAC_v2_gene31647	AT1G78750	1.2	NC	NC	protein.degradation.ubiquitin.E3.SCF.FBOX
Tp57577_TGAC_v2_gene6327	AT1G78750	1.3	1.2	NC	protein.degradation.ubiquitin.E3.SCF.FBOX
BGI_novel_G001317	AT4G00320	NC	1.1	NC	protein.degradation.ubiquitin.E3.SCF.FBOX
BGI_novel_G000041	AT5G56370	-1.3	NC	NC	protein.degradation.ubiquitin.E3.SCF.FBOX
BGI_novel_G000200	AT5G56810	5.3	NC	NC	protein.degradation.ubiquitin.E3.SCF.FBOX
BGI_novel_G000611	AT5G22730	1.7	1.4	NC	protein.degradation.ubiquitin.E3.SCF.FBOX
BGI_novel_G001155	AT1G80470	1.2	NC	NC	protein.degradation.ubiquitin.E3.SCF.FBOX
BGI_novel_G001356	AT4G26350	1.3	NC	NC	protein.degradation.ubiquitin.E3.SCF.FBOX
Tp57577_TGAC_v2_gene16816	AT3G52680	6.4	5.2	NC	protein.degradation.ubiquitin.E3.SCF.FBOX

Gene ID	Arabidopsis orthologs locus	Log2(RVY_ vs_RGY)	Log2(RV_ vs_RG)	Log2(RV_ vs_RVG)	BinName
Tp57577_TGAC_v2_gene24740	AT5G22700	-1.1	-1.2	NC	protein.degradation.ubiquitin.E3.SCF.FBOX
Tp57577_TGAC_v2_gene32739	AT5G56370	1.3	1.1	NC	protein.degradation.ubiquitin.E3.SCF.FBOX
Tp57577_TGAC_v2_gene35231	AT5G56370	2.2	2.4	NC	protein.degradation.ubiquitin.E3.SCF.FBOX
Tp57577_TGAC_v2_gene35235	AT5G56370	1.5	NC	NC	protein.degradation.ubiquitin.E3.SCF.FBOX
Tp57577_TGAC_v2_gene35236	AT5G56370	1.0	1.0	NC	protein.degradation.ubiquitin.E3.SCF.FBOX
BGI_novel_G000037	AT5G56370	NC	1.1	NC	protein.degradation.ubiquitin.E3.SCF.FBOX
Tp57577_TGAC_v2_gene21504	AT3G24760	-1.2	1.2	NC	protein.degradation.ubiquitin.E3.SCF.FBOX
Tp57577_TGAC_v2_gene24174	AT1G15670	1.7	NC	NC	protein.degradation.ubiquitin.E3.SCF.FBOX
Tp57577_TGAC_v2_gene24397	AT3G59940	-1.1	NC	NC	protein.degradation.ubiquitin.E3.SCF.FBOX
Tp57577_TGAC_v2_gene37271	AT1G15670	-1.9	NC	NC	protein.degradation.ubiquitin.E3.SCF.FBOX
Tp57577_TGAC_v2_gene24774	AT3G24760	NC	1.6	NC	protein.degradation.ubiquitin.E3.SCF.FBOX
Tp57577_TGAC_v2_gene3793	AT1G27340	NC	1.6	NC	protein.degradation.ubiquitin.E3.SCF.FBOX
Tp57577_TGAC_v2_gene38279	AT5G40680	NC	5.1	-1.2	protein.degradation.ubiquitin.E3.SCF.FBOX
BGI_novel_G000923	AT1G14330	NC	NC	-1.9	protein.degradation.ubiquitin.E3.SCF.FBOX
Tp57577_TGAC_v2_gene26543	AT5G24560	1.1	NC	NC	protein.degradation.ubiquitin.E3.SCF.FBOX
Tp57577_TGAC_v2_gene22114	AT2G02250	5.3	NC	NC	protein.degradation.ubiquitin.E3.SCF.FBOX
Tp57577_TGAC_v2_gene40740	AT2G02250	1.2	2.4	NC	protein.degradation.ubiquitin.E3.SCF.FBOX
BGI_novel_G000491	AT4G05497	-1.7	-1.5	NC	protein.degradation.ubiquitin.E3.SCF.FBOX
Tp57577_TGAC_v2_gene1659	AT5G01720	1.2	1.4	NC	protein.degradation.ubiquitin.E3.SCF.FBOX
Tp57577_TGAC_v2_gene20082	AT2G18280	6.6	7.1	NC	protein.degradation.ubiquitin.E3.SCF.FBOX
Tp57577_TGAC_v2_gene24444	AT2G03530	NC	-1.0	NC	protein.degradation.ubiquitin.E3.SCF.FBOX
Tp57577_TGAC_v2_gene28431	AT3G50080	NC	1.0	NC	protein.degradation.ubiquitin.E3.SCF.FBOX
Tp57577_TGAC_v2_gene39597	AT3G42830	NC	1.6	NC	protein.degradation.ubiquitin.E3.SCF.RBX
Tp57577_TGAC_v2_gene34156	AT5G42190	1.5	NC	NC	protein.degradation.ubiquitin.E3.SCF.SKP
Tp57577_TGAC_v2_gene10133	AT1G04810	-1.2	NC	NC	protein.degradation.ubiquitin.proteasom

Gene ID	Arabidopsis orthologs locus	Log_2(RVY_ vs_RGY)	Log_2(RV_ vs_RG)	Log_2(RV_ vs_RVG)	BinName
Tp57577_TGAC_v2_gene37079	AT4G28470	NC	-1.5	-1.1	protein.degradation.ubiquitin.proteasom
Tp57577_TGAC_v2_gene26137	AT3G26340	1.6	3.5	NC	protein.degradation.ubiquitin.proteasom
Tp57577_TGAC_v2_gene23951	AT1G53780	-3.6	-8.6	NC	protein.degradation.ubiquitin.proteasom
Tp57577_TGAC_v2_gene10997	AT5G20620	-4.2	-3.2	NC	protein.degradation.ubiquitin.ubiquitin
Tp57577_TGAC_v2_gene28042	AT5G20620	1.2	1.0	NC	protein.degradation.ubiquitin.ubiquitin
Tp57577_TGAC_v2_gene5	AT5G20620	NC	1.1	NC	protein.degradation.ubiquitin.ubiquitin
Tp57577_TGAC_v2_gene27489	AT4G02890	NC	1.4	NC	protein.degradation.ubiquitin.ubiquitin
Tp57577_TGAC_v2_gene21636	AT5G40630	2.9	NC	NC	protein.degradation.ubiquitin.ubiquitin
Tp57577_TGAC_v2_gene11596	AT3G45180	NC	2.5	NC	protein.degradation.ubiquitin.ubiquitin
Tp57577_TGAC_v2_gene14396	AT3G11910	-7.2	-6.2	NC	protein.degradation.ubiquitin.ubiquitin protease
Tp57577_TGAC_v2_gene1020	AT5G46740	1.5	NC	NC	protein.degradation.ubiquitin.ubiquitin protease

Note: NC, Log_2(Fold change) <|1| deemed no significant difference.

Table S3 Database information

Acronym	Details	Website
GO	Gene Ontology	http://geneontology.org
KEGG	Kyoto Encyclopedia of Genes and Genomes	http://www.genome.jp/kegg
NR/Nt	Non-redundant protein/nucleotide sequence database	ftp://ftp.ncbi.nlm.nih.gov/blast/db
Swiss-prot	Manually annotated and reviewed protein sequence database	http://www.gpmaw.com/html/swiss-prot.html
Pfam	Protein family	http://pfam.xfam.org
KOG	Clusters of orthologous groups of proteins	https://www.ncbi.nlm.nih.gov/COG/
PlantTFDB	Plant transcription factor database	http://plntfdb.bio.uni-potsdam.de/v3.0/
PRGdb	Plant resistance gene database	http://prgdb.crg.eu/

Table S4 Software and parameters used for data analysis

Software	Version	Parameter
SOAPnuke	v1.4.0	-l 15 -q 0.2 -n 0.1
HISAT2	v2.1.0	--dta --phred64 unstranded --new-summary -x index -1 read_r1 -2 read_r2 (PE)
Trimmomatic	v0.36	ILLUMINACLIP:2:30:10 LEADING:3 TRAILING:3 SLIDINGWINDOW:4:15 MINLEN:50
StringTie	v1.0.4	-f 0.3 -j 3 -c 5 -g 100 -s 10000 -p 8
Bowtie2	v2.2.5	-q --phred64 --sensitive --dpad 0 --gbar 99999999 --mp 1,1 --np 1 --score-min L,0,-0.1 -p 16 -k 200
RSEM	v1.2.8	Default value
CPC	v0.9-r2	Default value
DEGseq	——	Fold Change >= 2 and Adjusted Pvalue <= 0.001
R software	——	——
Mercator	v3.6	BLAST_CUTOFF: 80
MapMan	v3.6.0	Default value
Venn	V2.1	——
Tbtools	v1.098689	Default value

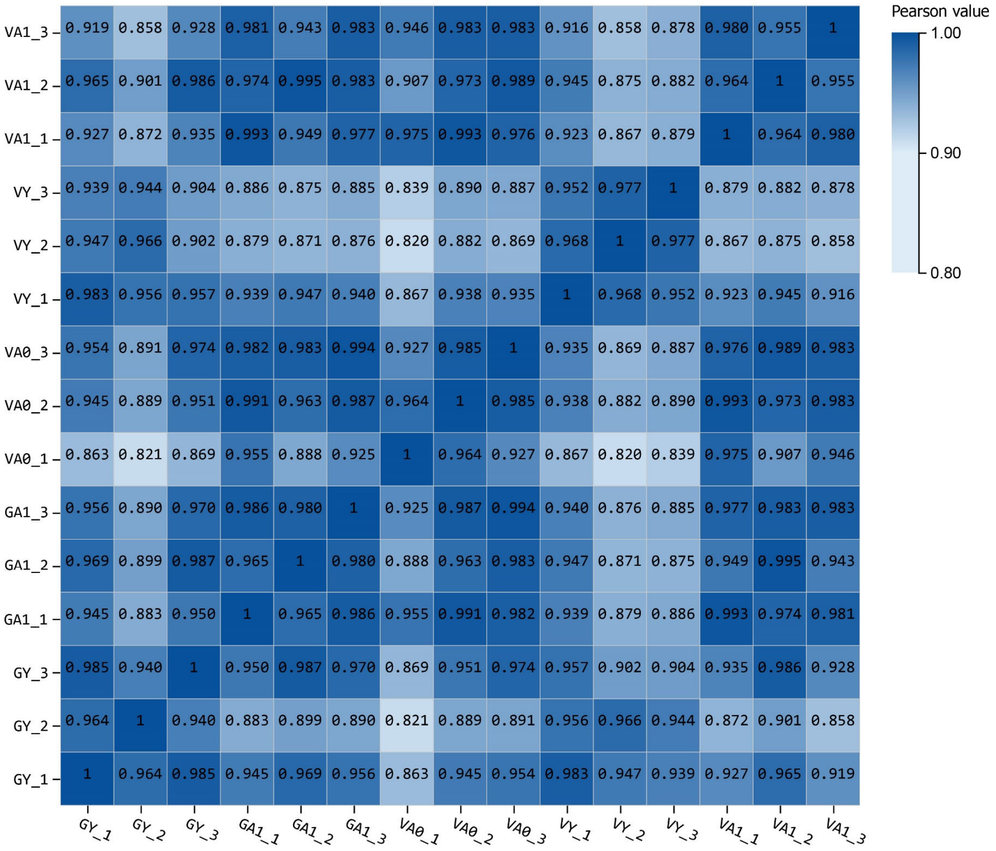

Figure S1. Pearson's correlation coefficient of RNA-seq data for leaflets of *Trifolium pratense*

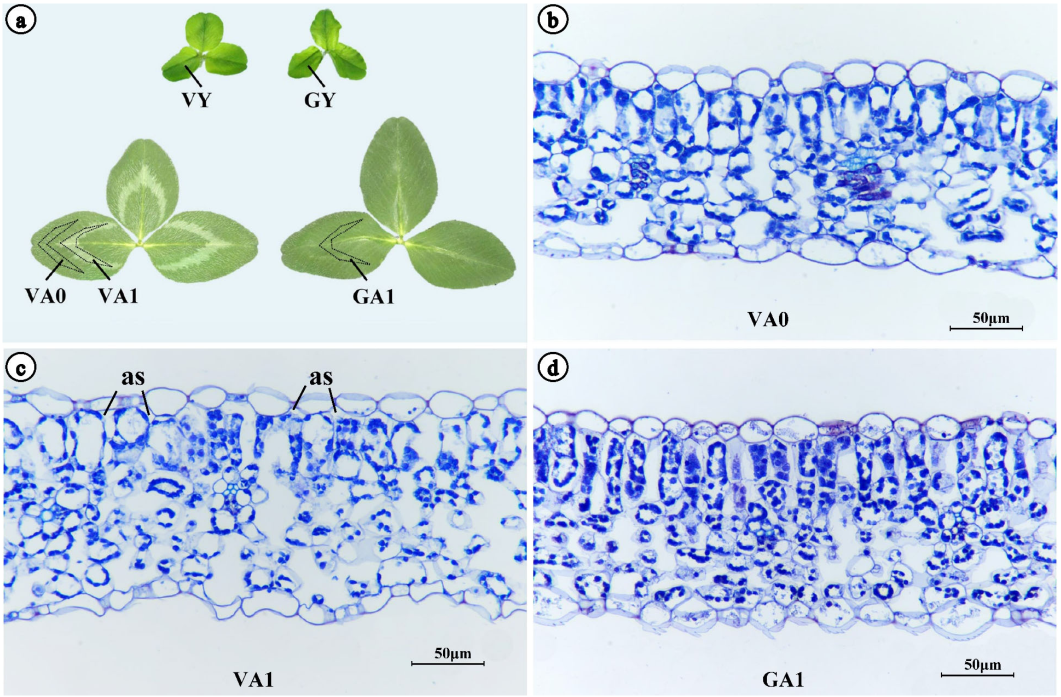

Figure S2. The sampling sites **(a)** and Comparative leaf structure **(b-d)** of *Trifolium pratense*. **GA**, green plants adult leaflets; **GY**, green plants young leaflets; **VA**, variegated plants adult leaflets; **VY**, variegated plants young leaflets; **0**, the area unable to be white; **1**, the white area on VA or the corresponding area on GA; **Dashed line**, sampling position

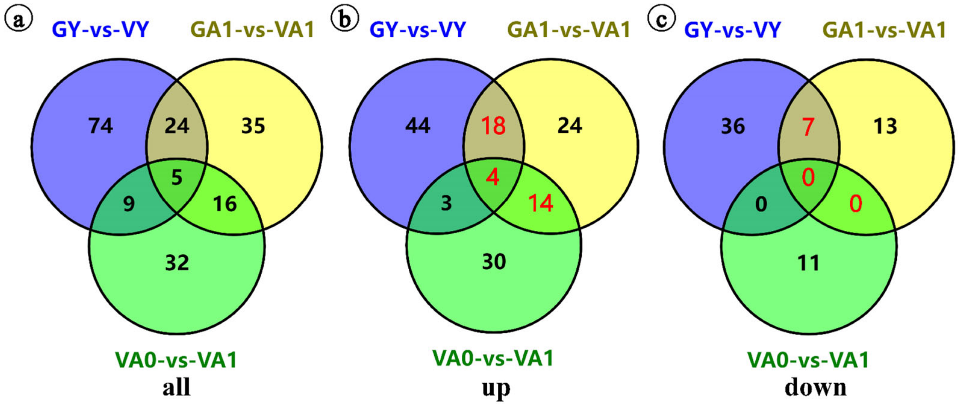

Figure S3. Venn analysis after MapMan analysis of the three comparison groups. **a**, total DEGs; **b**, up-regulated DEGs; **c**, down-regulated DEGs; **red numbers**, focused DEGs.

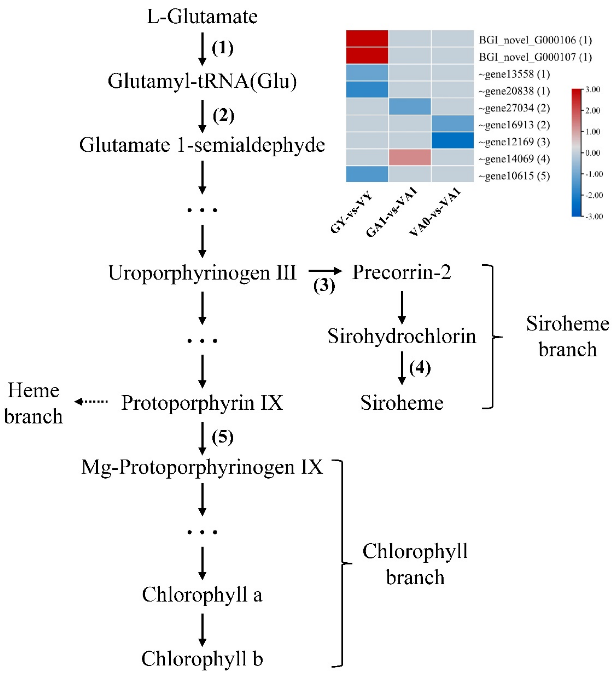

Figure S4. Tetrapyrrole metabolism of *Trifolium pratense*. **red**, up-regulated; **blue**, down-regulated.
